# The utility of accelerometers to predict stroke rate in captive fur seals and sea lions

**DOI:** 10.1242/bio.027029

**Published:** 2017-08-10

**Authors:** Monique A. Ladds, David A. Rosen, David J. Slip, Robert G. Harcourt

**Affiliations:** 1 Marine Predator Research Group, Department of Biological Sciences, Macquarie University, North Ryde, NSW 2113, Australia; 2 School of Mathematics and Statistics, Victoria University of Wellington, Wellington 6140, New Zealand; 3 Marine Mammal Research Unit, Department of Zoology, University of British Columbia, Vancouver, BC, V6T 1Z4, Canada; 4 Taronga Conservation Society Australia, Bradley's Head Road, Mosman, NSW 2088, Australia

**Keywords:** Otariid, Swim mechanics, Stroke rate, Accelerometer, Energetics, Biologger

## Abstract

The energy expenditure of free-living fur seals and sea lions is difficult to measure directly, but may be indirectly derived from flipper stroke rate. We filmed 10 captive otariids swimming with accelerometers either attached to a harness (Daily Diary: sampling frequency 32 Hz, *N*=4) or taped to the fur (G6a+: 25 Hz, *N*=6). We used down sampling to derive four recording rates from each accelerometer (Daily Diary: 32, 16, 8, 4 Hz; G6a+: 25, 20, 10, 5 Hz). For each of these sampling frequencies, we derived 20 combinations of two parameters (RMW, the window size used to calculate the running mean; and *m*, the minimum number of points smaller than a local maxima used to detect a peak) from the dynamic acceleration of x, z and x+z, to estimate stroke rate from the accelerometers. These estimates differed by up to ∼20% in comparison to the actual number of foreflipper strokes counted from videos. RMW and the choice of axis used to make the calculations (x, z or x+z) had little effect on the overall differences, though the variability was reduced when using x+z. The best *m* varied depending on the axis used and the sampling frequency; a larger *m* was needed for higher sampling frequencies. This study demonstrates that when parameters are appropriately tuned, accelerometers are a simple yet valid tool for estimating the stroke rates of swimming otariids.

## Abstract (precis)

**Summary:** Accelerometer data collected from captive fur seals and sea lions swimming were used to determine the best method for processing raw data to achieve the highest accuracy of stroke rate.

## INTRODUCTION

Measuring energy expenditure of pinnipeds is an important but difficult task. As swimming is a major source of energy expenditure, measuring stroke rate is a simple method to predict energy expenditure in otariid (Jeanniard-du-Dot et al., 2016) and phocid (Williams et al., 2004) seals. These two groups are evolutionarily divergent with completely different mechanics for underwater propulsion. Otariids propel themselves using a sculling motion of their large fore flippers (Feldkamp, 1987), while phocids rely on lateral movement of their hind flippers (Gallon et al., 2007). Despite these differences, stroke rate might have wide application as a proxy for energy expenditure (Williams et al., 2004).

Accelerometers are one means of estimating stroke rate, through measuring instantaneous acceleration forces in multiple axes. Estimating stroke frequency from accelerometers assumes that the peaks in the outputs correspond to a stroke. The lateral sway of the rear flippers of swimming phocid seals will result in strokes corresponding to peaks in the y-axis, as confirmed using animal borne cameras (Williams et al., 2004). Otariids use large fore-flippers to propel forward, therefore peaks in the surge (x-axis) and/or heave (z-axis) could potentially be used to estimate strokes (Jeanniard-du-Dot et al., 2016). However, the sensitivity of using accelerometers for estimating stroke rate in free-swimming pinnipeds has yet to be evaluated.

The ability to accurately delineate flipper strokes from accelerometry data depends on how the raw data are processed. For example, the choice of the width of the running mean and the choice of a minimum number of points smaller than a local maxima to detect a peak in the data influences the overall estimate (Shepard et al., 2008a). Therefore, the influence of these two parameters on the overall estimate must be tested across a range of values. Here, we evaluate the best parameters for calculating stroke rate for otariids swimming underwater by observing and filming animals swimming in aquariums while wearing accelerometers.

## RESULTS AND DISCUSSION

**Fig. 1.**
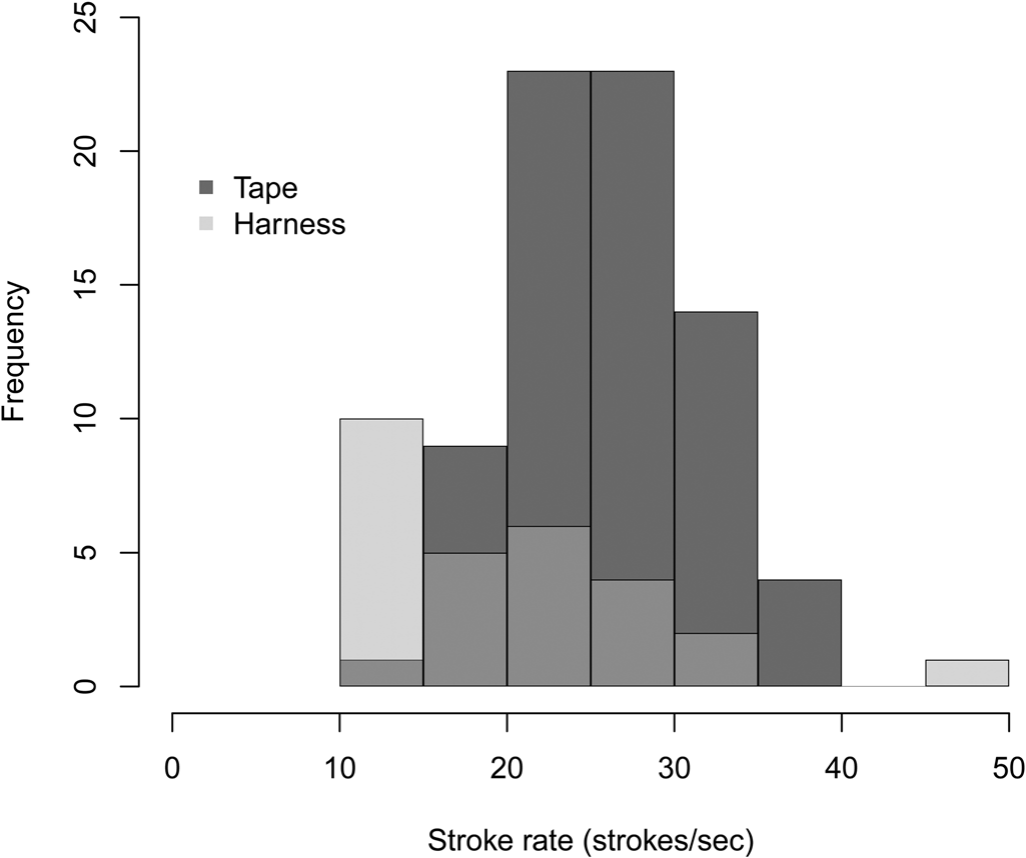
**Frequency distribution of actual flipper strokes counted from video footage for two groups of otariids swimming submerged.** The tape group had the accelerometer taped onto the fur, and the harness group had the accelerometer fitted to a custom-made harness.

**Fig. 2.**
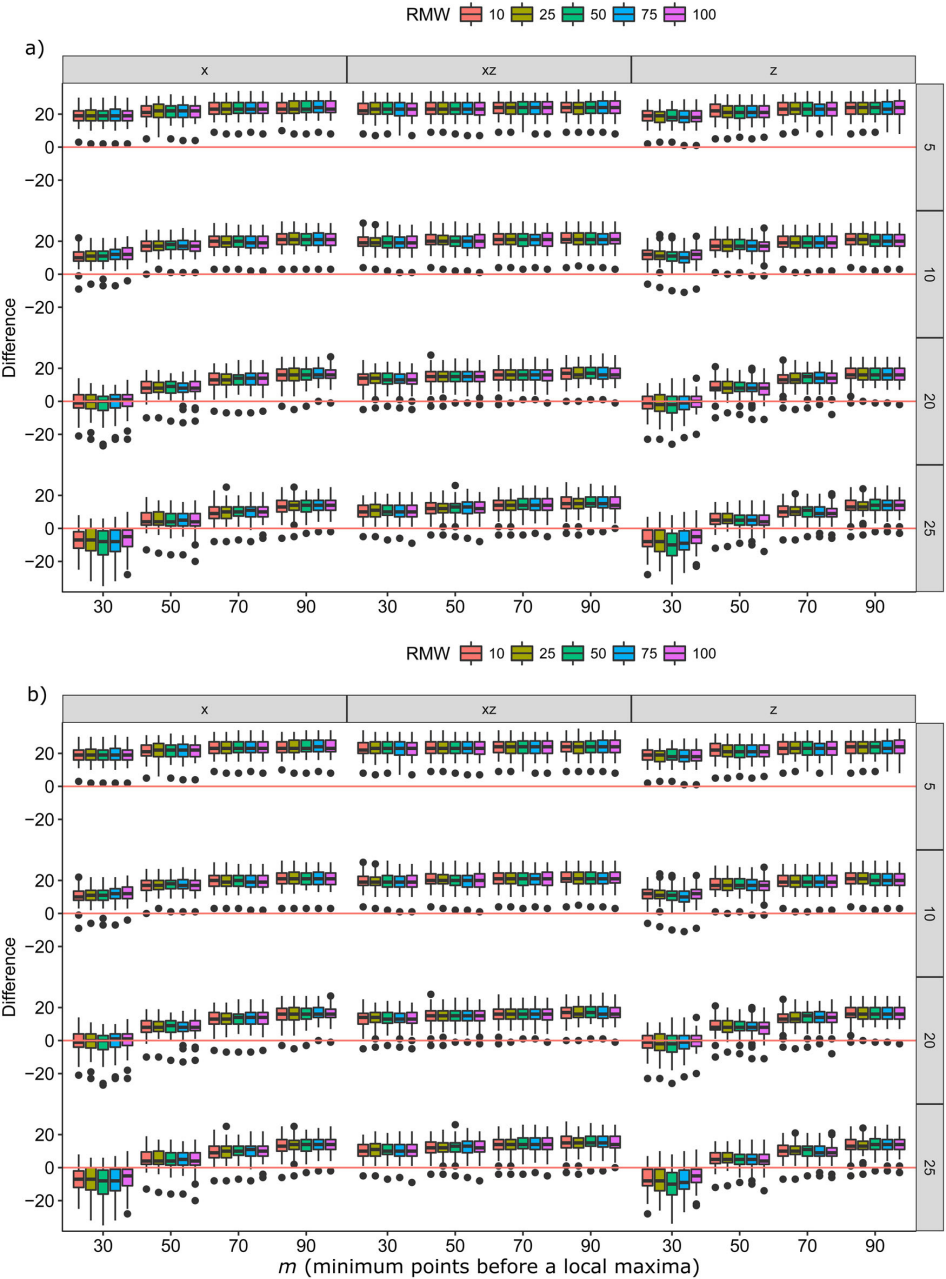
**Boxplots of differences (z-axis) between predicted stroke rate and actual stroke rate over different RMW (coloured boxes) and *m* (x-axis) for the three axes tested (x, z, x+z) and different sampling frequencies (HZ, y-axis).** Red lines indicate where there is 0 difference between observed strokes and predicted strokes. (A) Otariids with the accelerometer taped on (*N*=49 trials); (B) otariids wearing a harness with the accelerometer (*N*=71 trials).

The two groups tested displayed very different stroke patterns and frequencies. The harness group swam with intermittent bursts of swimming between feeding tubes, resulting in a much lower stroking frequency than the tape group, which swam constantly between target poles (Fig. 1). These different swimming styles and different sampling rates affected the parameters which best predicted stroke rate over a given dive. Stroke rate could be accurately predicted from finding peaks in the dynamic acceleration of all the axes tested (x, z and x+z axes), where no one axis was always better at predicting stroke rate than another (Fig. 2). The exact combinations of RMW (the window size used to calculate the running mean) and *m* (the minimum number of points smaller than a local maxima used to detect a peak) required to achieve the lowest error rates for stroke prediction differed between individuals (Table 1) and across groups (Fig. 2). The absolute mean difference between the number of strokes predicted and the number of actual strokes for each dive across both groups was between −0.97 and 1.97, which represented a percentage difference of between −4.42% and 7.47% (Table 1).

**Table 1.**
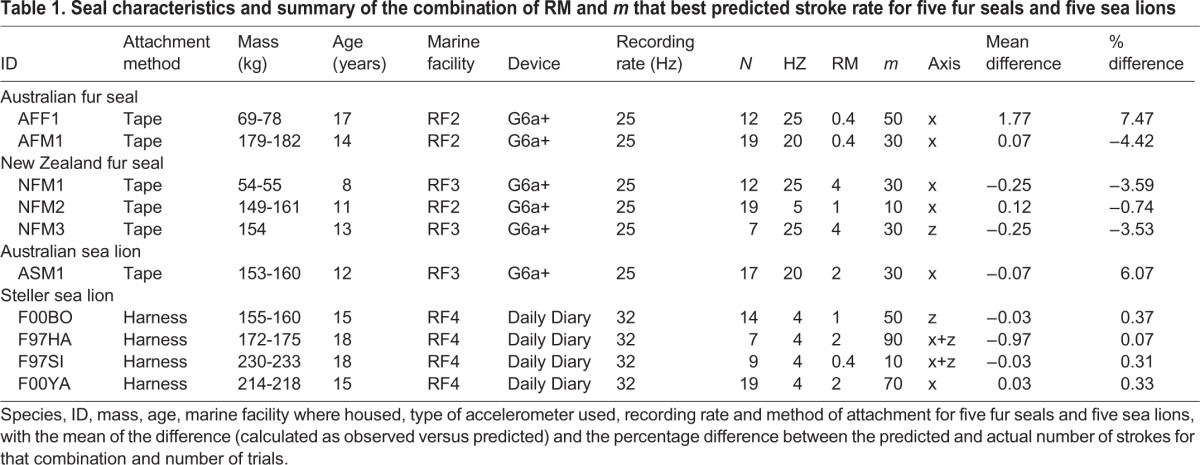
**Seal characteristics and summary of the combination of RM and *m* that best predicted stroke rate for five fur seals and five sea lions**

Fig. 2 displays the distribution of errors for all combinations of axis, sampling frequency, RMW and *m* tested. Distributions centred on 0, with small variances representing the best combinations, which differed for each group. The best combination was chosen from the distribution that was not significantly different from 0 (observed=estimated) as determined by *t*-tests. The accuracy of predictions depended on both the sampling frequency and *m* (Fig. 2). Performing *t*-tests revealed that 25 of the combinations of RMW, *m*, sampling frequency and axis tested were not significantly different from 0 (Fig. 2). For the tape group, the combination that produced the least errors was using a sampling rate of 20, with an *m* of 30, and calculated from either the x or z axis (Fig. 2A). There was less consistency in the predictions for the harness group. If a sampling rate of 16 Hz was used then an *m* of 50 was best using the z or x axis, but if 32 Hz was used then an *m* of 90 using the x+z axis produced the least errors. Altering the RMW to smooth the data had very little influence on the predictions of either group. Accelerometry data consist of only peaks and troughs; therefore, it is important to choose an *m* that corresponds only to a stroke and not to other movement of the body. Choosing the correct combination of running mean and gradient to predict stroke rate is important because the total number of strokes could be under- or overpredicted by ∼20%. Given that the optimal parameters for the two groups were vastly different, it is important that each parameter is selected with careful tuning when deploying accelerometers on wild seals.

Accelerometers measure the movement of an animal in three directions – surge, heave and sway – and the amplitudes of these measurements are dependent on the activity of the animal. The stroke pattern of otariids causes a distinct surge of acceleration forward (x-axis) and upward (z-axis), which, in theory, can be used to calculate stroke rate (Jeanniard-du-Dot et al., 2016). Here, we have shown that these steep peaks can be identified using a simple peak detection parameter (*m*), but that selecting *m* that results in the fewest errors depends on the sampling frequency of the accelerometer and the axis chosen to estimate the stroke rate. For otariids that have the accelerometer attached with tape, an *m* of 30 or 50 could be used to detect strokes. For otariids with the accelerometer placed in a harness, a larger *m* helped to account for the additional data points generated from the higher sampling rate of the accelerometer (32 Hz versus 25 Hz) and movement from the harness. As dynamic body acceleration (DBA) is derived from applying a running mean over the axes of acceleration, in theory the window size (RMW) used to calculate the running mean changes the value of the DBA, and therefore the ability of DBA to predict stroke rate and ultimately estimate energy expenditure (Shepard et al., 2008a). However, in this study, the RMW chosen did not have a large influence on the overall prediction of stroke rate. Instead the sampling frequency used and the *m* selected were more important in detecting strokes accurately.

While the miniaturisation of data loggers is making it easier to collect data from free-living animals, data storage can still be an issue, particularly if the goal is to monitor the animal over a long period. We found little variation between the ability of a single axis (x or z) compared to a combination of axes (x+z) to predict stroke rate. This suggests that if memory or power of a logger limits long-term deployments, it is still possible to obtain good stroke rate measures using only the x-axis. Sampling frequency may also affect the ability to predict stroke rate from accelerometers as lower rates of sampling have been shown to be more variable (Halsey et al., 2009). However, here we demonstrated that reducing the sampling frequency reduced the variation in the predictions. Reducing the sampling frequency to 20 Hz for the tape group and to 16 Hz for the harness group significantly increased the number of accurate stroke predictions (Fig. 2). Potentially this means that in wild studies a much lower sampling frequency could be used, saving battery and memory of devices, allowing them to be deployed for much longer durations (Halsey et al., 2009).

Stroke rate may be a useful proxy for estimating energy expenditure (Jeanniard-du-Dot et al., 2016), but as with any proxy, it is important that steps are taken to validate its utility. Here, we attached accelerometers, recording at different frequencies, to fur seals and sea lions swimming under controlled conditions. We found that recording frequency and the *m* used to estimate strokes were the most important parameters in detecting peaks (identified as strokes from video analysis) recorded from the accelerometer. Further, the RMW and axis selected had little influence on the accuracy of the estimated number of strokes. Therefore, when using accelerometers to estimate stroke rate for otariids, any running mean of between 0.4 and 4 s is appropriate, measured on a single axis (x or z). However, the *m* used must be selected according to the sampling frequency of the accelerometer, where a larger *m* is required for a higher sampling frequency. This study shows that accelerometers are a simple yet valid tool for estimating the stroke rates of swimming otariids, provided care is taken in selecting the appropriate gradient for identifying peaks in the accelerometry.

## MATERIALS AND METHODS

### Animals

We conducted experiments during October to December 2014 at two research facilities, Underwater World (Mooloolaba, Australia; RF2) and Taronga Zoo (Sydney, Australia; RF3), with three New Zealand fur seals (*Arctocephalus forsteri*), two Australian fur seals (*Arctocephalus pusillus*) and four Australian sea lions (*Neophoca cinerea*) that were on permanent display at their respective marine facilities. We conducted experiments in November and December 2015 at the University of British Columbia's Open Water Research Station (Port Moody, Canada; RF4) with four Steller sea lions (*Eumetopias jubatus*) housed for research (Table 1). All animals were nonreproductive during the study period and cared for under individual facility husbandry guidelines. All animals were assessed to be in good health and condition at the time of the experiments. Macquarie University ethics committee (ARA-2012_064) and Taronga Zoo ethics committee (4c/10/13) approved the experiments in Australia (the experiments were authorized under a New South Wales Office of Environment and Heritage Scientific Licence, SL100746). In Canada, handling and experimental procedures were in accordance with regulations of the Canadian Council on Animal Care (University of British Columbia animal use permit A11-0397), Department of Fisheries and Oceans Canada (MML 2007-001) and the Vancouver Aquarium.

### Trial protocol

During experiments, the seals were equipped with one of two types of three-axis accelerometer (at RF2/3: CEFAS G6a+, ±8 g, 12-bit resolution; 40×28×16.3 mm, and mass 18 g in air and 4.3 g in seawater, CEFAS Technology Ltd, Lowestoft, UK; or at RF4: Daily Diary, ±2 g, resolution 0.05 m/s/s, 95×45×26 mm, 90 g, Wildlife Computers, Redmond, USA; Table 1). At RF2/3, the G6a+ accelerometer was taped (Tesa, Hamburg Germany) between the shoulder blades, resulting in very little movement of the accelerometer because of close attachment to the body. At RF4, sea lions wore a custom-made harness with the Daily Diary secured to the outside. Although trainers tightened the harness to closely fit the body of the sea lion, this attachment method resulted in more movement of the accelerometer as it was not attached directly to the fur. Sea lions at RF4 had been trained to swim underwater in the open ocean between two submerged feeding stations (Rosen et al., 2016) while seals at RF2/3 were trained to swim laps of a pool between two stationary targets (Ladds et al., 2016). All animals were familiar with the experimental equipment and performed all trials voluntarily under trainer control.

### Stroke rate estimation

Accelerometers (G6a+ and Daily Diaries) recorded time, depth, and acceleration on three axes: anterior-posterior (surge, x-axis), lateral (sway, y-axis) and dorso-ventral (heave, z-axis). Video footage allowing us to directly count stroke rate was pseudo-randomly collected from 10 animals at RF2, RF3 and RF4 while participating in other experiments (Ladds et al., 2016). Underwater swimming at RF2 and RF3 was recorded with a GoPro (GoPro Hero3 Black edition, 1080p/Wide/60 fps) mounted inside PVC pipes, each with a viewing window cut-out (see Hocking et al., 2015 for a figure of the set up). For sea lions at RF4, the GoPro was mounted on their harness and oriented towards the pectoral flipper. Videos were downloaded and edited together in Adobe Premiere Pro (Adobe Systems, California, USA), before being exported at the same frame rate as the accelerometer recorded (i.e. G6a+ 25 FPS and 25 Hz at RF1-3; Daily Diary 32 FPS and 32 Hz at RF4). Accelerometer data were matched with the corresponding time stamp on the video, allowing us to extract data for dives. The actual total number of strokes for a trial was counted from videos of individual trials, where a stroke was counted if a cycle of movement of the flipper was completed. Strokes that used a single flipper or that were only below the body were not included as they were often masked on the accelerometry by other movement. These movements could not be counted consistently across the different groups as the orientation of the GoPro for the sea lions at RF4 restricted view of the entire stroke.

### Statistical analysis

**Fig. 3.**
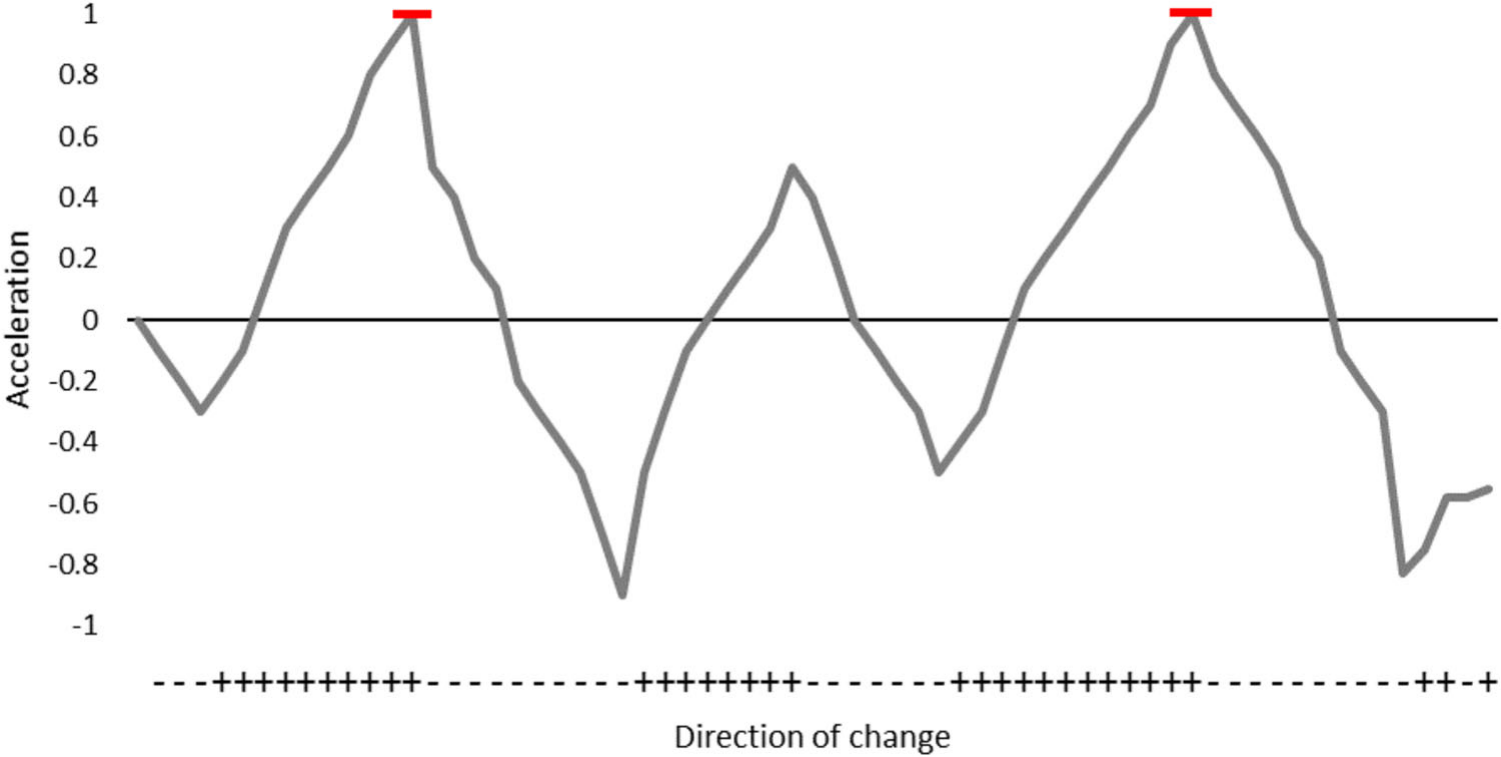
**Example of how strokes are detected in the accelerometry with an *m* of 10.** If 10 consecutive positive differences either side of a peak in the dynamic acceleration are detected then a stroke is counted. If more than 10 positive or negative differences are detected, then the stroke is identified at the end of the run of positive differences.

As recording rate and attachment method of the accelerometers differed, the analysis was carried out for two groups. The first group had a G6a+ accelerometer recording at 25 Hz taped to the fur (referred to as the tape group) and the second group had a Daily Diary recording at 32 Hz fitted on a harness (referred to as the harness group). To test how different sampling frequencies affected the overall stroke prediction, data from each accelerometer were downsampled to 32, 16, 8 and 4 Hz for the Daily Diary, and 25, 20, 10 and 5 Hz for the G6a+. DBA required to estimate strokes, is derived from applying a running mean over the axes of acceleration to calculate static acceleration (gravity) and removing this from the raw acceleration (Shepard et al., 2008b). The accelerometry data were first smoothed using a running mean (of various lengths, see below) to remove the effect of gravity and create dynamic acceleration. The dynamic acceleration was then used to predict stroke rate, where strokes were identified as peaks in the accelerometry data. A peak is defined as the local maximum, where a minimum of *m* points either side of it are smaller. For example, if the minimum *m* is set to 10, then there must be at least 10 consecutive smaller values before and after a maximum (peak) to be marked as a stroke (Fig. 3). As stroke rate can be estimated from peaks in the dynamic acceleration of the x, z or x+z (adding the dynamic acceleration of x and z together) axes, all options were tested. In addition, both the size of the window used to calculate the running mean (hereafter RMW) and the minimum number of smaller points before a peak (hereafter *m*) of the peaks influenced the overall stroke rate estimate, so combinations of these two variables were created. The RMW tested were 0.4, 1, 2, 3 and 4 s and the *m* tested were 30, 50, 70 and 90. Gradients were chosen from preliminarily data exploration examining visually which gradients detected the peaks in the dynamic acceleration. The best stroke rate prediction was defined as the running mean and gradient that resulted in the fewest errors when compared to observed stroke rates. This was determined by testing if the differences were significantly different from 0, meaning no difference in observed and measured strokes, using a one-sample *t*-test.

All analyses were completed in R (Version 3.1.3) (R Core Development Team, 2015) and values are reported as mean±s.d.
